# Rotavirus epidemiology and genotype distribution in hospitalised children, Greece, 2008 to 2020: A prospective multicentre study

**DOI:** 10.2807/1560-7917.ES.2022.27.47.2101133

**Published:** 2022-11-24

**Authors:** Dimitra-Maria Koukou, Athanasios Michos, Panagiota Chatzichristou, Georgios Trimis, Elizabeth-Barbara Tatsi, Charilaos Dellis, Levantia Zachariadou, Theodota Liakopoulou, George P Chrousos, Vasiliki Syriopoulou, on behalf of Greek Rotavirus Study Group

**Affiliations:** 1 First Department of Pediatrics, Medical School, National and Kapodistrian University of Athens, ‘Aghia Sophia’ Children’s Hospital, Athens, Greece; 2 MSD Greece, Medical and Scientific Affairs Department, Athens, Greece; 3 University Research Institute of Maternal and Child Health and Precision Medicine, Athens, Greece; 4 Microbiology Department, ‘Aghia Sophia’ Children’s Hospital, Athens, Greece; 5 First Department of Pediatrics, Iaso Children’s Hospital, Athens, Greece; 6 Members of the Greek rotavirus study group are listed under Acknowledgements

**Keywords:** Rotavirus, genotypes, children, gastroenteritis, Greece

## Abstract

**Background:**

Two rotavirus (RV) vaccines were licensed in Greece in late 2006 and included in the national immunisation programme in 2012.

**Aim:**

To study the epidemiology and genotype distribution of RV in children during the post-vaccination period and assess the impact of increased vaccination coverage.

**Methods:**

In a prospective multicentre hospital-based study, hospitalised children (≤ 16 years) with an RV-positive faecal sample were recruited. Epidemiological and genotyping analyses were performed; periods of low (2008–12) and moderate (2012–20) RV vaccination coverage were compared. Statistical analysis was performed with a chi-squared or Mann–Whitney U test and logistic regression.

**Results:**

A total of 3,874 children (55.6% male; n = 2,153) with median age of 1.4 years (IQR: 0.5–3.3) were studied during 2008–20. Most RV-infected children were aged ≤ 3 years (72.2%) and hospitalised during December–May (69.1%). Common RV genotypes (G1P[8], G2P[4], G3P[8], G4P[8], G9P[8], G12P[8]) were detected in 92.2% of samples; G-P combinations with prevalence above 1% were G4P[8] (44.1%), G1P[8] (25.4%), G2P[4] (14.9%), G9P[8] (3.5%), G12P[8] (2.2%), G3P[8] (2.1%), other (4.3%) and mixed (3.5%). Of all samples, 97.6% were homotypic or partially heterotypic to vaccines’ genotypes. With moderate vaccination coverage, the seasonal peak was detected earlier, children were older and partially or fully heterotypic genotypes were increased (p < 0.001).

**Conclusions:**

In the era of moderate RV vaccination coverage in Greece, epidemiology of RV in hospitalised children seemed to change. However, most circulating genotypes remain homotypic or partially heterotypic to RV vaccines. Continuous epidemiological surveillance and genotyping are important to monitor possible changes arising from RV vaccines’ implementation.

**Key public health message**

**What did you want to address in this study?**

Rotavirus (RV) vaccines protect against RV infection, which is a major cause of diarrhoea in children. We wanted to investigate how the epidemiology of RV infection changed in Greece since the introduction of RV vaccines in late 2006.

**What have we learnt from this study?**

Since RV vaccines were introduced in Greece, changes in seasonal, geographical and age distribution of RV infection are reported. Most of the circulating genotypes (97.6%) are homotypic or partially heterotypic to vaccines’ genotypes and there is no indication of the emergence of RV genotypes that could evade immunity afforded by vaccination.

**What are the implications of your findings for public health?**

Current epidemiology and genotype distribution of RV in Greece is consistent with data from other European countries. With moderate RV vaccination coverage in Greece, changes in the RV epidemiology have emerged and current vaccines cover circulating genotypes. Continuous surveillance is important to draw solid conclusions about the effect of the vaccines on the RV epidemiology.

## Introduction

Rotavirus (RV) is the leading cause of acute gastroenteritis (GE) in young children and a major public health problem worldwide [1]. Vaccination is considered a primary measure to control RV infection and reduce related morbidity and mortality. In 2006, two oral, live-attenuated RV vaccines were licensed: RotaTeq (Merck), a three-dose, pentavalent vaccine including human genotypes (G1, G2, G3, G4, P[8]) reassorted into the bovine WC3 strain (G6P7[5]) and Rotarix (GlaxoSmithKline), a two-dose, monovalent vaccine derived from an attenuated human G1P[8] strain [2,3]. In 2013, the World Health Organization (WHO) recommended that RV vaccines should be introduced in all national immunisation programs [4]. By January 2022, RV vaccination has been implemented in over 110 countries worldwide [5].

Before RV vaccination was available in Greece, RV caused 20–50% of viral GE cases among children who sought medical evaluation, depending on the seasonal peak of RV [6-8]. In late 2006, both RV vaccines were licensed in Greece, but were initially only available through private paediatricians at the patient’s expense. RV vaccination coverage in Greece remained low (< 25%) during the first 5 years after the vaccines’ release on the private market [9] and observational studies conducted during this period in Greece reported a decline in the burden of rotavirus gastroenteritis (RVGE) in children aged < 5 years [10,11]. By 2012, when both RV vaccines had been introduced into the national immunisation programme (NIP) with partial funding, RV vaccination coverage was gradually increased [12]. According to vaccines’ sales data from 2021, current vaccination coverage has reached moderate levels (55%) for both RV vaccines in children aged under 12 months. 

RV vaccine development was based on the homotypic or heterotypic immune response to the most common human RV genotypes [13,14]. Αs known, group A RVs (RVA) show great genetic diversity and are classified into G and P genotypes depending on two outer capsid viral proteins (VP) VP7 and VP4, respectively. The most prevalent genotypes are: G1P[8], G2P[4], G3P[8], G4P[8], G9P[8] and G12P[8] [15]. Although RV genotype distribution differs both geographically and temporally, the implementation of RV vaccination worldwide has brought concern about the effect of the vaccines on the global RV genotyping distribution and the emergence of newly or reassortant strains through mechanisms of possible selection pressure [14]. 

The aims of this study were to describe the epidemiology of RV and the distribution of RV genotypes in hospitalised children with RVGE infection in Greece during consecutive years in the post-vaccination period (2008–20), as well as to assess whether the gradual increase of RV vaccination coverage affected the RV epidemiology and genotype distribution.

## Methods

### Study setting

In January 2007, the European Rotavirus Network ‘EuroRotaNet’ was established to study the molecular epidemiology of RV infection and monitor the emergence and spread of common and novel RV strains within Europe during the post-vaccination era, collecting data from different European countries [16]. Greece became a member of the EuroRotaNet in January 2009; current membership of EuroRotaNet includes 13 European countries [17]. 

A Greek RV study group consisting of 20 paediatric hospitals was created in September 2008 to monitor children with RVGE and analyse RV positive faecal samples according to EuroRotaNet’s guidelines. Collaborating paediatric hospitals were located in 10 of 13 geographical regions of Greece, which serve 93.1% of the paediatric population both in urban and rural areas [18]. For reasons of the statistical analysis, paediatric hospitals were further divided into northern (n = 10), central (n = 7) and southern (n = 3), according to their geographical setting (Supplementary Figure S1 shows the geographical setting of paediatric hospitals in the Greek rotavirus study group).

### Study design

A prospective multicentre hospital-based study was conducted during the period from September 2008 to August 2020. The RV year was defined as the period from September to the following August. Every RV year contained four RV seasons: winter (December to February), spring (March to May), summer (June to August) and autumn (September to November). The RV seasonal peak was defined as the month with the highest number of RV specimens. The time periods for analysis were further divided into periods of low vaccination coverage (September 2008–August 2012) and moderate vaccination coverage (September 2012–August 2020), based on the year of introduction of the two RV vaccines into the NIP, and the reported levels of vaccination coverage. 

### Case definition and sample collection

A RV case was defined as a hospitalised child aged ≤ 16 years who presented symptoms of GE (≥ 3 watery stools/day or doubling the normal number of bowel movements/day combined or not with vomiting and/or fever) and had a faecal sample positive for RVA antigen which was available for genotyping. 

The RVA antigen detection was performed as part of laboratory active surveillance testing in the microbiology department of the collaborating paediatric hospitals with a rapid immunochromatographic test (VIKIA Rota-Adeno test, bioMérieux). Maximum time from sample collection to antigen testing was 8 h. Positive RV faecal samples were stored properly at 2–8°C according to EuroRotaNet’s protocol and were shipped within a 10-day period for genotyping to the paediatric infectious diseases and chemotherapy ‘Choremeio’ research laboratory of the University of Athens. The study’s minimum annual target number of RV samples was set by EuroRotaNet’s protocol and enabled detection of RV genotypes with prevalence of ≥ 1% [17]. Demographic data (e.g. sex, age, address), RV vaccination status and date of sample collection were also collected from each child whose faecal sample was sent for genotyping.

### Rotavirus genotyping

Positive faecal samples were prepared with Stool Transport and Recovery buffer (Roche Diagnostics). The viral RNA genome was extracted employing the MagNA Pure Compact Nucleic Acid Isolation Kit I (Roche Diagnostics) on the MagNA Pure Compact instrument according to the manufacturer's instructions. Viral RNA was stored at −80°C or immediately used in the reverse transcription process. Synthesis of cDNA was carried out using the Transcriptor First Strand cDNA Synthesis Kit (Roche Diagnostics) according to the manufacturer's instructions. 

A multiplex semi-nested PCR of the VP7 and VP4 genes was conducted using the GoTaq DNA Polymerase (Promega) and specific primers according to the EuroRotaNet’s rotavirus detection and typing methods [17]. The PCR products were characterised as G (G1–12) and P (P4–11) types according to their size by a 2% agarose gel electrophoresis using a 50 bp DNA ladder (New England Biolabs). Samples that could not be genotyped by gel electrophoresis were analysed by Sanger sequencing with the BigDye Terminator v3.1 Cycle Sequencing Kit on an Applied Biosystems 3500 Genetic Analyzer (Applied Biosystems). The electrochromatographic data from sequencing were further analysed with BLAST.

Further categorisation of RV genotypes was performed. Samples were characterised as ‘common’ if they belonged to one of the six common human strains (G1P[8], G2P[4], G3P[8], G4P[8], G9P[8], G12P[8]) based on previous epidemiological studies in Europe [15,16,19]. If they did not belong to a common strain, they were characterised as ‘other’. If they contained more than one G- or/and P-type, they were categorised as ‘mixed’. Partially typeable or non-typable faecal samples were excluded from the genotyping analysis. 

RV genotypes were characterised as ‘homotypic’ if they had both G-type and P-type antigen similarity to the RV vaccines’ genotypes (G1, G2, G3, G4, P[8]), ‘fully heterotypic’ if they had no G-P antigen similarity to the vaccines’ genotypes and ‘partially heterotypic’ if they had G or P antigen similarity to the vaccines’ genotypes [14].

### Statistical analyses

Statistical analyses were performed with the statistical software package IBM SPSS statistics v25 (SPSS, Inc.). Categorical data were expressed as absolute numbers and proportions (%). The chi-squared and Fisher-exact tests were used to assess the correlation between categorical variables and to compare categorical variables between periods of low and moderate vaccination coverage. Continuous data were tested for normality using One-Sample Kolmogorov–Smirnoff test and graphical methods, and were reported as mean ± standard deviation (SD) in case of normal distribution or median and interquartile range (IQR) in case of non-normal distribution. Mann–Whitney U test was performed to assess differences of the quantitative variables between two groups. Binary logistic regression was used to predict the correlation between periods of low and moderate vaccination coverage and a set of predictor variables. A p value of < 0.05 was considered statistically significant.

## Results

A total of 3,874 hospitalised children with RVGE were enrolled in the study, including 1,815 children during the period of low vaccination coverage and 2,059 children during the period of moderate vaccination coverage. The total RV vaccination rate of hospitalised children with RVGE was 1.2% (46/3,874) and there was no significant difference in vaccination rates between the periods of low and moderate vaccination coverage (1.0% and 1.3% respectively).

### Demographic and geographic distribution

Of the hospitalised children with RVGE, 2,153 (55.6%) were male; the sex ratio did not differ between the periods of low and moderate vaccination coverage (p = 0.119). The median age of the study population was 1.4 years (IQR: 0.5–3.3). The median age of hospitalised children with RVGE during the period of moderate vaccination coverage was 1.6 years (IQR: 0.5–3.6 years), which was higher than the median age of children during the low vaccination coverage (1.3 years; IQR: 0.5–2.7 years; p < 0.001). The predominant age group of children with RVGE was ≤ 1 year of age (39.1%). Children aged ≤ 3 years represented 72.2% of the study’s population. A comparison between age groups of children between the two periods revealed that percentage of children aged ≤ 3 years was lower during the period of moderate vaccination coverage while percentage of children aged > 3 years was higher (p < 0.001) (**Table 1**).

**Table 1:** Characteristics of children aged ≤ 16 years hospitalised with rotavirus gastroenteritis during periods of low and moderate rotavirus vaccination coverage, Greece, 2008–2020 (n = 3,874)

Variables		Total n = 3,874		Vaccination coverage				p value
				Low (2008–12) n = 1,815		Moderate (2012–20) n = 2,059		
		n	%	n	%	n	%	
Sex	Male	2,153	55.6	990	54.5	1,163	56.5	0.119
	Female	1,721	44.4	825	45.5	896	43.5	
Age (years)	Median (IQR)	1.4	0.5–3.3	1.3	0.5–2.7	1.6	0.5–3.6	< 0.001
Age group (years)	≤ 1	1,515	39.1	753	41.5	762	37.0	< 0.001
	> 1–2	806	20.8	410	22.6	395	19.2	
	> 2–3	476	12.3	229	12.6	249	12.1	
	> 3–6	744	19.2	341	18.8	402	19.5	
	> 6–16	333	8.6	82	4.5	251	12.2	
Season	Winter	1,345	34.7	655	36.1	690	33.5	< 0.001
	Spring	1,330	34.4	663	36.5	667	32.4	
	Summer	601	15.5	275	15.2	326	15.8	
	Autumn	598	15.4	222	12.2	376	18.3	
Geographical region	Northern	460	11.9	343	18.9	117	5.7	< 0.001
	Central	3,232	83.4	1,323	72.9	1,909	92.7	
	Southern	182	4.7	149	8.2	33	1.6	

IQR: interquartile range.

P value refers to a chi-squared test between periods of low and moderate rotavirus vaccination coverage vs demographic/epidemiological variables. A p value of < 0.05 was considered statistically significant.

Most of the children lived in urban areas (3,281/3,874; 84.7%); 11.9% (n = 460), 83.4% (n = 3,232) and 4.7% (n = 182) lived in the northern, central and southern geographical regions of Greece, respectively (**Table 1**). The distribution of hospitalised children with RVGE per paediatric hospital showed that most children (2,601/3,874; 67.1%) were hospitalised at the ‘Aghia Sophia’ Children's Hospital in Athens, which is the largest tertiary paediatric hospital in Greece. Supplementary Table S1 shows the distribution of children per paediatric hospital. During the period of moderate vaccination coverage, the percentage of children with RVGE living in northern and southern regions of Greece was significantly lower (**Table 1**).

### Seasonal distribution

**Figure 1:**
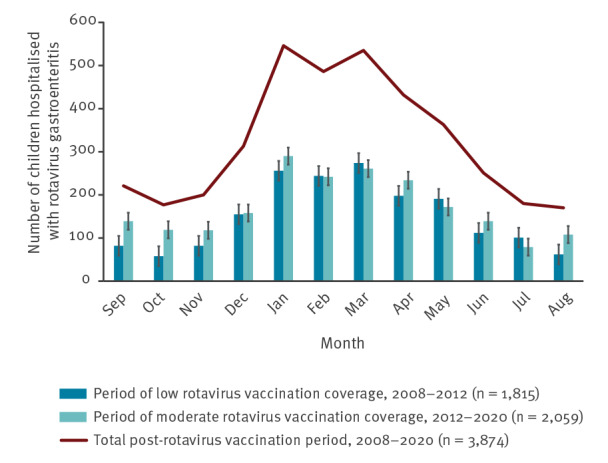
Monthly distribution of children aged ≤ 16 years hospitalised with rotavirus gastroenteritis during the post-rotavirus vaccination period, Greece, 2008–2020 (n = 3,874)

Although hospitalised children with RVGE were detected throughout the year, 69.1% (2,675/3,874) were detected in winter and spring (December to May) (**Table 1**). Comparing the seasonal distribution during periods of low and moderate vaccination coverage, we found that during the period of moderate vaccination coverage, the percentage of children with RVGE who were detected in summer and autumn was increased, while the percentage of children who were detected in winter and spring was decreased (p < 0.001) (**Figure 1**). The RV seasonal peak was observed in January followed by a second peak in March. Comparing the periods of low and moderate vaccination coverage, the RV seasonal peak was significantly different (p < 0.001). In the low coverage period, the RV seasonal peak was observed in March and in the moderate coverage period in January (p < 0.001 for both periods).

Error bars indicate the standard error of the number of children hospitalised with rotavirus gastroenteritis.

### Genotype distribution

RV genotypes were identified in 86.4% (3,346/3,874) of faecal samples. The remaining 528 samples were only partially or not genotyped because of inadequate amount or possible limitations of the current genotyping methods. The distribution of RV genotypes were common human strains in 92.2% (3,085/3,346) of the samples (G4P[8] (44.1%), G1P[8] (25.4%), G2P[4] (14.9%), G9P[8] (3.5%), G12P[8] (2.2%), G3P[8] (2.1%)). Other and mixed genotypes were found in 4.3% and 3.5% of the samples, respectively (**Table 2**)**.** Genotype comparison to RV vaccines’ genotypes showed that 73.8% (n = 2,468) of the sample strains were homotypic, 23.8% (n = 799) were partially heterotypic, and 2.4% (n = 79) were fully heterotypic (**Table 2**).

**Table 2:** Distribution of rotavirus genotypes in children aged ≤ 16 years hospitalised with rotavirus gastroenteritis during periods of low and moderate rotavirus vaccination coverage, Greece, 2008–2020 (n = 3,346)

Genotype		Total n = 3,346		Vaccination coverage				p value
				Low (2008–12) n = 1,446		Moderate (2012–20) n = 1,900		
		n	%	n	%	n	%	
G-P type combinations	G1P[8]	849	25.4	410	28.4	439	23.1	0.001
	G2P[4]	497	14.9	227	15.7	270	14.2	
	G3P[8]	71	2.1	45	3.1	26	1.4	
	G4P[8]	1,476	44.1	637	44.1	839	44.2	
	G9P[8]	117	3.5	31	2.1	86	4.5	
	G12P[8]	75	2.2	39	2.7	36	1.9	
	Other^a,c^	143	4.3	32	2.2	111	5.8	
	Mixed^b,c^	118	3.5	25	1.7	93	4.9	
Similarity to RV vaccines’ genotypes	Homotypic	2,468	73.8	1,100	76.1	1,368	72.0	0.001
	Partially heterotypic	799	23.8	328	22.7	471	24.8	
	Fully heterotypic	79	2.4	18	1.2	61	3.2	

RV: rotavirus.

^a^ Other genotypes, see Box.

^b^ Mixed genotypes, see Box.

^C^ In the period of low vaccination coverage, G9P[4] and G1+G4P[8] were not detected, while in the period of moderate vaccination coverage, these genotypes were detected in 1.5% (50/3,346) and 1.3% (44/3,346) of the total samples, respectively.

P value refers to a chi-squared test between periods of low and moderate vaccination coverage vs RV genotypes. A p value < 0.05 was considered statistically significant.

Other genotypes consisted of 25 G-P combinations and were either a reassortment of common human strains or reassortment between human and animal strains or strains with possible animal origin (Box). They were isolated in 6 different areas of Greece (Athens, Kalamata, Karditsa, Lamia, Larisa and Volos). Mixed genotypes consisted of 21 different G-P combinations and contained more than one G-type, or more than one P-type or more than one G- and P-type (Box). They were isolated in 8 different areas of Greece (Athens, Kalamata, Veroia, Volos, Larisa, Karditsa, Thessaloniki and Arta).

Box **Rotavirus G-P combinations of other genotypes and mixed genotypes, Greece, 2008–2020**

**Other genotypes**

Reassorted common human strains: G1P[4], G2P[8], G3P[4], G4P[4], G9P[4]

Reassortment between human and animal strains or strains with possible animal origin: G1P[9], G2P[10], G2P[6], G3P[9], G4P[10], G4P[14], G4P[6], G4P[9], G6P[14], G6P[9], G8P[14], G8P[8], G9P[10], G9P[9], G10P[4], G10P[8], G12P[11], G12P[4], G12P[6], G12P[9]

**Mixed genotypes**

More than one G-type: G1+G2P[4], G1+G3P[9], G1+G4P[8], G1+G9P[8], G1+G12P[8], G2+G4P[4], G2+G4P[8], G4+G12P[4], G4+G12P[8], G4+G9P[8], G8+G9P[8]

More than one P-type: G1P[8]+P[4], G10P[8]+P[10], G2P[8]+P[4], G2P[8]+P[10], G3P[8]+P[4], G4P[8]+P[4], G4P[8]+P[9], G9P[8]+P[6]

More than one G- and P-type: G2+G4P[8]+P[4], G4+G1P[8]+P[6]

Comparing genotypes between periods of low and moderate vaccination coverage, it was found that G4P[8] was the predominant genotype in both periods (44.1% and 44.2%, respectively). Genotypes G1P[8] and G2P[4] were the second and third most frequently detected types in both periods, followed by G3P[8] and G12P[8] in the first period and G9P[8] in the second period. Other and mixed genotypes were detected more often in the second period (p < 0.001). Especially G9P[4] and G1 + G4P[8] were not detected at all during the first period and were detected during the second period in 1.5% (50/3,346) and 1.3% (44/3,346) of the total samples, respectively. Strains with genotypes partially or fully heterotypic to the RV vaccines’ strains were also detected more often during the second period (p < 0.001) **(** **Table 2** **).**

**Figure 2:**
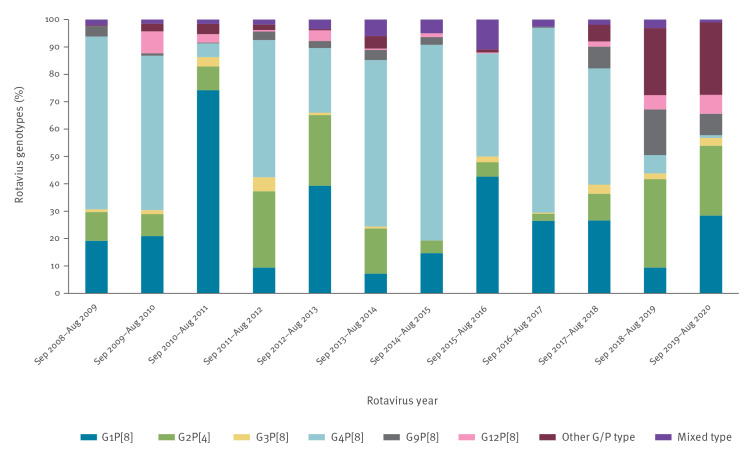
Annual distribution of rotavirus genotypes circulating in children aged ≤ 16 years hospitalised with rotavirus gastroenteritis during the post-rotavirus vaccination period, Greece, 2008–2020 (n = 3,346)

The annual (September to August) distribution of RV genotypes varied (**Figure 2** **).** During the first decade of the study (2008–18), the predominant genotypes were either G4P[8] (42.5–71.5%) or G1P[8] (39.3–74.2%). During the last 2 years of the study, the predominant genotypes were different. In 2018–19, G2P[4] was the predominant genotype (32.3%) followed by other genotypes (mostly G9P[4]; 16.7%) that were detected in 24.5% of the samples. In the same year, G9P[8] was significantly increased (16.7%). In 2019–20, G1P[8] was the predominant genotype (28.4%), followed by other genotypes (mostly G9P[4]; (9.8%)) that were detected in 26.5% of the samples and G2P[4] (25.5%). The rest of the genotypes showed significant annual variations and some were not isolated at all during specific years, like G3P[8] in 2014–15 and G9P[8] in 2015–16. The emerging strain G12P[8] appeared for the first time in 2009–10 with a frequency of 8%; afterwards, it was detected in low percentages (0.5–6.9%) every year with the exception of 2016–17, when it was not detected at all. Other genotypes were detected during all the study apart from two periods (2014–15 and 2016–17). Mixed genotypes were detected during the entire study period with frequencies of 1.0–10.9%. The annual distribution of RV genotypes in numbers is presented in Supplementary Table S2.

Other and mixed genotypes are listed in the Box.

### Genotype and demographic/epidemiological variables

No correlation was found between genotype and sex (p = 0.108) **(** **Table 3** **).** Genotypes G9P[8] and G12P[8] were detected more often in children aged > 1–2 years and the rest of genotypes in infants aged ≤ 1 year (p < 0.005). Genotypes G2P[4], G3P[8] and G4P[8] were more frequent in winter and all the other genotypes in spring (p < 0.001). Regarding geographical regions, genotype G4P[8] was dominant in northern and central Greece, while G1P[8] was dominant in southern Greece (p < 0.001). 

**Table 3:** Statistical correlation between demographic/epidemiological variables and rotavirus genotypes detected in children aged ≤ 16 years hospitalised with rotavirus gastroenteritis during the post-rotavirus vaccination period, Greece, 2008–2020 (n = 3,346)

Variables		Genotype																										p value
		G1P[8] n = 849			G2P[4] n =497			G3P[8] n = 71			G4P[8] n =1,476			G9P[8] n = 117			G12P[8] n = 75			Other^a^ n = 143			Mixed^a^ n = 118			Total n = 3,346		
		n	%	n		%	n		%	n		%	n		%	n		%	n		%	n		%	n		%	
Sex	Male	475	55.9	258		51.9	42		59.2	824		55.8	63		53.8	44		58.7	90		62.9	78		66.1	1,874		56.0	0.108
	Female	374	44.1	239		48.1	29		40.8	652		44.2	54		46.2	31		41.3	53		37.1	40		33.9	1,472		44.0	
Age group (years)	≤ 1	312	36.7	191		38.4	33		46.5	534		36.2	32		27.4	19		25.3	57		39.9	53		44.9	1,231		36.8	0.005
	> 1–2	188	22.1	104		20.9	15		21.1	289		19.6	35		29.9	25		33.3	29		20.3	13		11.0	698		20.9	
	> 2–3	110	13.0	60		12.1	4		5.6	209		14.2	17		14.5	10		13.3	14		9.8	18		15.3	442		13.2	
	> 3–6	166	19.6	95		19.1	13		18.3	332		22.5	23		19.7	13		17.3	21		14.7	22		18.6	685		20.5	
	> 6–16	73	8.6	47		9.5	6		8.5	112		7.6	10		8.5	8		10.7	22		15.4	12		10.2	290		8.7	
Season	Winter	268	31.6	210		42.3	36		50.7	611		41.4	29		24.8	24		32.0	20		14.0	30		25.4	1,228		36.7	0.001
	Spring	343	40.4	163		32.8	22		31.0	480		32.5	46		39.3	30		40.0	49		34.3	45		38.1	1,178		35.2	
	Summer	121	14.3	60		12.1	6		8.5	211		14.3	27		23.1	14		18.7	29		20.3	27		22.9	495		14.8	
	Autumn	117	13.8	64		12.9	7		9.9	174		11.8	15		12.8	7		9.3	45		31.5	16		13.6	445		13.3	
Geographical region	Northern	90	23.2	64		16.5	13		3.4	172		44.3	22		5.7	10		2.6	6		1.5	11		2.8	388		100	0.001
	Central	699	25.0	396		14.2	54		1.9	1251		44.8	92		3.3	61		2.2	133		4.8	106		3.8	2,792		100	
	Southern	60	36.1	37		22.3	4		2.4	53		31.9	3		1.8	4		2.4	4		2.4	1		0.6	166		100	

P value refers to a chi-squared test between genotype vs demographic/epidemiological variables. A p value < 0.05 was considered statistically significant.

^a^ Other and mixed genotypes are listed in the Box.

### Periods of low and moderate rotavirus vaccination coverage and demographic/epidemiological variables

In the period of moderate vaccination coverage, hospitalised children with RVGE aged > 6–16 years were more prevalent compared with children ≤ 1 year of age (p < 0.001). Children with RVGE were detected more often during summer (p = 0.021) and autumn (p < 0.001) compared with winter, and lived more often in central geographical region compared with northern regions (p < 0.001). Additionally, during the same period, G4P[8] (p = 0.012), G9P[8] (p < 0.001), other (p < 0.001) and mixed genotypes (p < 0.001) were detected more often compared with G1P[8], while G3P[8] was detected significantly less often compared with G1P[8] (p = 0.040) (**Table 4**).

**Table 4:** Binary logistic regression between periods of low and moderate rotavirus vaccination coverage and demographic/epidemiological variables for children aged ≤ 16 years hospitalised with rotavirus gastroenteritis during the post-rotavirus vaccination period, Greece, 2008–2020 (n = 3,874)

Variables		β	p value	OR	95% CI
Sex	Male	Ref.			
	Female	−0.067	0.378	0.935	0.806–1.085
Age group (years)	≤ 1	Ref.			
	> 1–2	−0.120	0.242	0.887	0.725–1.084
	> 2–3	−0.102	0.395	0.903	0.714–1.142
	> 3–6	−0.088	0.389	0.916	0.750–1.119
	> 6–16	0.878	0.001	2.405	1.747–3.310
Season	Winter	Ref.			
	Spring	0.127	0.154	1.135	0.954–1.351
	Summer	0.270	0.021	1.310	1.041–1.648
	Autumn	0.481	0.001	1.618	1.265–2.067
Geographical region	Northern	Ref.			
	Central	1.411	0.001	4.099	3.193–5.262
	Southern	−0.350	0.130	0.705	0.448–1.109
Genotype^a^	G1P[8]	Ref.			
	G2P[4]	0.192	0.116	1.211	0.954–1.538
	G3P[8]	−0.554	0.040	0.575	0.339–0.975
	G4P[8]	0.234	0.012	1.263	1.053–1.516
	G9P[8]	1.262	0.001	3.533	2.163–5.770
	G12P[8]	−0.144	0.574	0.866	0.524–1.430
	Other ^b^	1.005	0.001	2.732	1.755–4.251
	Mixed ^b^	1.175	0.001	3.240	1.985–5.286

β; unstandardised coefficient; CI: confidence interval; OR: odds ratio; Ref.: reference.

^a^ Genotype analysis was performed on 3,346 faecal samples since 528 samples were partially or not genotyped.

^b^ Other and mixed genotypes are listed in the Box.

A p value < 0.05 was considered statistically significant.

## Discussion

We performed a prospective multicentre hospital-based study on RV epidemiology and distribution of circulating genotypes in hospitalised children with RVGE during the era of RV vaccines’ implementation in Greece, and we found changes in the seasonal, geographical, age and genotype distribution as vaccine coverage increased.

The seasonal peaks of RVGE in Greece during the entire study period occurred in January and March. Typically, the RVGE seasonal peak begins in early winter in south-western Europe and appears in the eastern and northern Europe during early spring [16]. A similar pattern of spread from the south-west to the north-east has also been described in the United States (US) [20]. There are different theories regarding the seasonality of RV infection. In previous decades, Cook et al. demonstrated that RVGE had a seasonal peak during winter in countries with temperate climate and was present throughout the year in tropical countries [21]. More recent studies have attributed the RV seasonality to the nature of the virus and the socioeconomic level of the countries. For example, countries with lower income and higher birth rates like those in Africa, Asia and South America, display a lack of RV seasonality because of the high transmission rates of RV [22]. However, Patel et al. showed that there can be more than one factor that contributes to the pattern of RV seasonality. The socioeconomic level of a country can be a strong predictor of seasonal intensity but at a regional level, many factors may interact and explain seasonality including climate, transmission patterns, host behaviour and susceptibility [23].

Additionally, RV can cause asymptomatic infection or remain in the environment for a long time, resulting in seasonal epidemics when conditions favour its easy and rapid transmission [23]. In this study, RV samples were detected not only during winter and spring but also during autumn and summer, which could be attributed to small epidemic bursts of RVGE cases. Comparing the seasonality of RVGE during the periods of low and moderate vaccination coverage in Greece, we found that in the second period children with RVGE were detected more often in summer and autumn and the seasonal peak shifted and occurred earlier in January. In contrast, a shift to a later peak of RVGE occurred in the US and Europe after the implementation of RV vaccines [24,25]. It remains unclear yet whether these findings are attributed to the vaccines or to the natural seasonal fluctuations of RV.

Only 1.2% of the study’s population had received at least one dose of any RV vaccine. It has been reported that RV vaccines protect from severe RVGE, so it is expected that most of the children who present severe symptoms of RVGE and are hospitalised, like the children of our study, would be unvaccinated. In an RV epidemiology study from Finland where the RV vaccination coverage is up to 95%, Markkula et al. reported a higher percentage of vaccinated children with RVGE (9.6%) [26]. This percentage is higher compared to our study because in Finland RV vaccination coverage is higher and also the study was conducted in all clinical laboratories of the country and every detected RV strain was collected and genotyped during the period’s study. In our study, the collection of RV samples was not performed in all clinical laboratories of the country.

The age group with the highest percentage of RVGE was ≤ 1 year. The RVGE age distribution is explained by the fact that infants older than 3 months of age no longer have an immune boost from maternal antibodies and are more vulnerable to RV [27]. Moreover, during the period of moderate vaccination coverage, a significant increase in the median age of children with RVGE was found. According to the findings of the Global Surveillance Network’s meta-analyses, during the pre-vaccine period the median age of RVGE cases was 12 months (IQR: 7–20), whereas after vaccine introduction the median age was 15 months (IQR: 9–25; p < 0.0001) [28]. Similar observations have been reported separately by countries like the US, United Kingdom (UK), Belgium and Brazil, where an increase in the average age of children with RVGE and a decrease in the incidence of RVGE in infants aged < 1 year has been observed after the vaccines’ implementation [29-32].

A review of global RV strain prevalence data documented that the dominant six genotypes (G1P[8], G2P[4], G3P[8], G4P[8], G9P[8] and G12P[8]) have been isolated in 97% of the 14,438 collected samples from 25 European countries [15]. In the present study, the dominant six genotypes were detected in 92.2% of the samples. According to EuroRotaNet’s report for the period 2006–20 the six common genotypes together with the emerging G9P[4] made up 91% of all characterised strains [17]. Interestingly, a review of circulating genotypes in neighbouring Türkiye showed that the five common genotypes accounted only for 59.7% [33]. Apart from the six common genotypes, we detected other genotypes at a total rate of 4.3%. These genotypes were isolated sporadically, mostly in children aged ≤ 1 year during spring and autumn. Different combinations of the five common genotypes (G1P[4], G2P[8], G3P[4], G4P[4], G9P[4]) have been detected in several parts of the world at a relatively high frequency, indicating that these strains have genetic stability and possibly the ability to spread to the population [34]. Other less common G-P combinations with possible origin of recombination between human and animal strains have been detected sporadically in European countries, and are able to create small epidemics in closed populations, especially during out-of-peak seasons, as in our study [16,35]. Mixed genotypes were found in 3.5% of Greek samples, while in EuroRotaNet’s report, this number reached up to 8.6% and varied from country to country. Specifically, mixed RV genotypes reached 12% of the total samples in Italy and Austria, and 18% in Spain [17]. According to our findings, mixed genotypes had annual variations (1.0–10.9%) and increased significantly during 2015–16. Although mixed strains are not predominant and usually lead to epidemiological dead-ends, they should be taken into consideration in epidemiological studies, because as recombinant strains may play a key role in the evolution of RV. 

In our study, 97.6% of all genotypes were homotypic or partially heterotypic to the RV vaccines’ strains and during the moderate vaccination coverage period, partially or fully heterotypic genotypes were significantly increased. Specifically, during this period there was an increase in G9P[8], mixed and other genotypes. Among other genotypes, G9P[4] was significantly increased between September 2018 and August 2020. From March 2020 to August 2020, the coronavirus disease (COVID-19) pandemic was present and various measures such as social distancing, school closures and improved hand hygiene led to the decrease in the incidence of viral gastroenteritis [36]. Currently, there are no reports of new or emerging RV genotypes during the COVID-19 pandemic [17]. Nevertheless, RV genotype fluctuations have been reported in the previous years. According to the EuroRotaNet’s report, G9P[8] increased in 2015–16 (19–34% of all samples) and in 2019–20 was the predominant genotype in Finland (52%). Also G9P[4] was increased (5–7% of characterised samples) during 2017–20 in many European countries (UK, Belgium, Denmark, Austria, Slovenia, Sweden) [17]. A similar increase of G9P[4] has been reported by non-European countries like Argentina during the post vaccination period [37]. According to EuroRotaNet’s data, countries like the UK that use the monovalent vaccine have reported decline of G1P[8] and shift to heterotypic P[4] types (G2P[4] and G9P[4]) during the post vaccination era. However, other European countries (Germany, Belgium, Austria, Spain) have reported an increase in detection of G3P[8] in addition to an increase of P[4] types, regardless of the type of RV vaccine in use [17]. Thus, the emergence of heterotypic strains, such as G9P[4], should be interpreted with caution and not be attributed exclusively to the RV vaccine implementation.

In the present study, correlation of RV genotypes with the age of children was observed. Genotypes G9P[8] and G12P[8] were more common among older children. Association between genotype and age has been reported by the EuroRotaNet [35]. G2P[4] has been found to be more common in older children and especially in adults [26]. This can be explained by different genetic origin of G2P[4] (DS-1-like genotype constellation) compared with the genetic origin of other common genotypes (Wa-like genotype constellation). The different genotype constellation – and the fact that it is heterotypic to the RV vaccines – enables G2P[4] to circulate in older children (aged ≥ 6 years) and adults, many of whom are probably not vaccinated and have a different immune status compared with young children and infants [16,34]. In Greece, while there are no data on genotype distribution in adults, no increase in the frequency of G2P[4] in older children was reported. However, an increase of the heterotypic genotypes was observed in the older children.

Our study had some limitations. One limitation was the lack of measurement of RVGE annual incidence and trend analysis during the study period, as the study was designed according to the aim of the EuroRotaNet’s protocol, which is RV genotyping surveillance. Also, the collection of faecal samples and the testing for RV antigen were performed according to the protocol of each paediatric hospital, as there is no standard protocol nationwide or a national reporting system for viral gastroenteritis in Greece. Thus, some smaller paediatric hospitals across the country collected fewer RV faecal samples for genotyping. Another limitation was that we did not study the association between genotypes and clinical severity of RVGE cases, because study’s population consisted only of hospitalised children, who probably present more severe clinical symptoms.

## Conclusion

This multicentre RVGE epidemiological study in Greece confirms that RV epidemiology and genotype distribution differ geographically and temporally. A high percentage of RV genotypes are common and homotypic or partially heterotypic to the RV vaccines’ strains. During the period of moderate vaccination coverage there was an increase in partially or fully heterotypic genotypes, which was previously described by other European countries. It is important to continue RV epidemiological surveillance and molecular genotyping to draw conclusions about the effect of RV vaccination on the pattern of RV infection in relation to age, region and season and the possibility of emergence of new RV strains.

## Supplementary Data

336000 Supplement
