# Supplementary material for: Rotavirus epidemiology and genotype distribution in hospitalised children, Greece, 2008 to 2020: A prospective multicentre study
Source: Euro Surveill. 2022 Nov 24;27(47):2101133. doi: 10.2807/1560-7917.ES.2022.27.47.2101133 (PMC9693793; doi:10.2807/1560-7917.ES.2022.27.47.2101133)
Supplement: Supplement [file 21-01133_KOUKOU_SUPPLEMENT.pdf]

SUPPLEMENTARY MATERIAL

This supplementary material is hosted by *Eurosurveillance* as supporting information alongside the article **"Rotavirus epidemiology and genotype distribution in hospitalised children, Greece, 2008 to 2020: A prospective multicentre study",** on behalf of the authors, who remain responsible for the accuracy and appropriateness of the content. The same standards for ethics, copyright, attributions and permissions as for the article apply. Supplements are not edited by *Eurosurveillance* and the journal is not responsible for the maintenance of any links or email addresses provided therein.

Supplementary Figure S1. Geographical setting of paediatric hospitals that participated in the Greek rotavirus study group, Greece, 2008-2020.

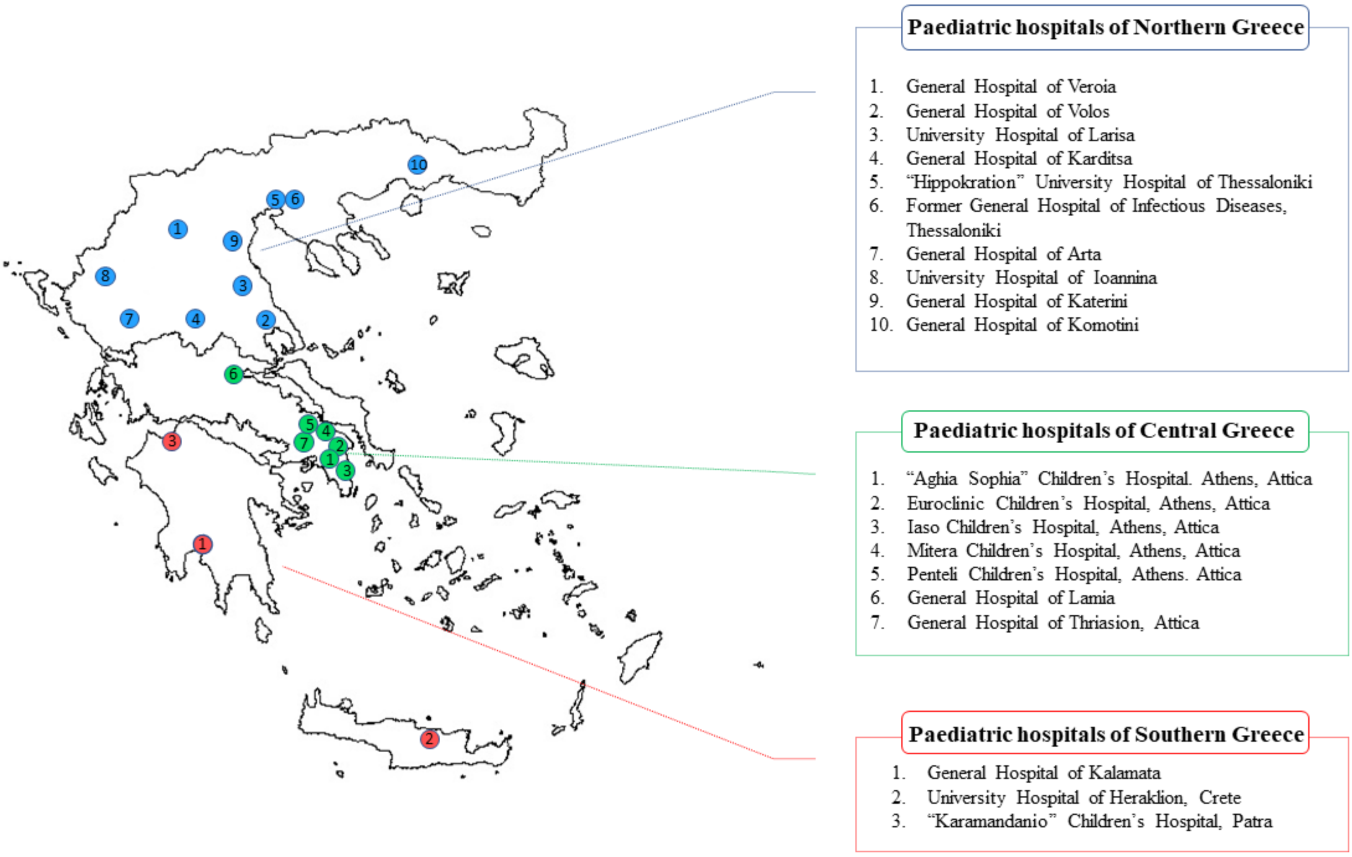

Supplementary Table S1. Distribution of children aged ≤ 16 years hospitalised with rotavirus gastroenteritis per paediatric hospital during the post-rotavirus vaccination period, Greece, 2008-2020 (n=3874).

**Paediatric hospitals**	**Number of children with RVGE**	**Percentage of children with RVGE**	**Cumulative Percentage of children with RVGE**
	**n**	**%**	**%**
‘Aghia Sophia’ children’s hospital. Athens, Attica	2601	67.1	67.1
Euroclinic children’s hospital, Athens, Attica	255	6.6	73.7
Iaso children’s hospital, Athens, Attica	146	3.8	77.5
Mitera children’s hospital, Athens, Attica	143	3.7	81.2
General hospital of Kalamata	122	3.1	84.3
General hospital of Veroia	117	3.0	87.3
General hospital of Volos †	83	2.1	89.4
University hospital of Larisa	71	1.8	91.2
General hospital of Karditsa †	47	1.2	92.4
‘Hippokration’ university hospital of Thessaloniki †	44	1.1	93.5
University hospital of Heraklion, Crete †	42	1.1	94.6
Penteli children’s hospital, Athens. Attica †	38	1.0	95.6
Former general hospital of infectious diseases, Thessaloniki †	36	0.9	96.5
General hospital of Lamia †	32	0.8	97.3
General hospital of Arta *	31	0.8	98.1
‘Karamandanio’ children’s hospital, Patra †	19	0.5	98.6
General hospital of Thriasion, Attica *	16	0.4	99.0
University hospital of Ioannina *	15	0.4	99.4
General hospital of Katerini*	10	0.3	99.7
General hospital of Komotini*	6	0.3	100.0
**Total**	3874	100.0	

RVGE: rotavirus gastroenteritis

* Paediatric hospitals that participated in the Greek rotavirus study group for the period 2008-2011.

† Paediatric hospitals that participated in the Greek rotavirus study group for the period 2008-2013.

Table is not representative of RVGE incidence.

Supplementary Table S2. Annual distribution of rotavirus genotypes circulating in children aged ≤16 years hospitalized with rotavirus gastroenteritis during the post-rotavirus vaccination period, Greece, 2008-2020 (n=3346).

**Rotavirus genotype***	**Sep.2008-Aug.2009**		**Sep.2009-Aug.2010**		**Sep.2010-Aug.2011**		**Sep.2011-Aug.2012**		**Sep.2012-Aug.2013**		**Sep. 2013-Aug. 2014**		**Sep. 2014-Aug.2015**		**Sep.2015-Aug.2016**		**Sep.2016-Aug.2017**		**Sep.2017-Aug.2018**		**SEep2018-Aug.2019**		**Sep.2019-Aug.2020**		**Total**	
	**n**	**%**	**n**	**%**	**n**	**%**	**n**	**%**	**n**	**%**	**n**	**%**	**n**	**%**	**n**	**%**	**n**	**%**	**n**	**%**	**n**	**%**	**n**	**%**	**n**	**%**
**G1P[8]**	55	19.2	68	20.9	239	74.2	48	9.4	90	39.3	31	7.2	32	14.7	121	42.6	61	26.5	57	26.6	18	9.4	29	28.4	849	100
**G2P[4]**	30	10.5	26	8.0	28	8.7	143	27.9	59	25.8	71	16.5	10	4.6	15	5.3	6	2.6	21	9.8	62	32.3	26	25.5	497	100
**G3P[8]**	3	1.0	5	1.5	11	3.4	26	5.1	2	0.9	3	0.7	0	0.0	6	2.1	1	0.4	7	3.3	4	2.1	3	2.9	71	100
**G4P[8]**	181	63.1	183	56.4	16	5.0	257	50.1	54	23.6	262	60.8	156	71.5	107	37.6	155	67.5	91	42.5	13	6.8	1	1.0	1476	100
**G9P[8]**	11	3.8	3	0.9	1	0.3	16	3.1	6	2.6	16	3.7	6	2.8	0	0.0	1	0.4	17	7.9	32	16.7	8	7.8	117	100
**G12P[8]**	0	0.0	26	8.0	10	3.1	3	0.6	9	3.9	2	0.5	3	1.4	1	0.4	0	0.0	4	1.9	10	5.2	7	6.9	75	100
**Other†**	1	0.3	9	2.8	12	3.7	10	2.0	1	0.4	20	4.6	0	0.0	3	1.1	0	0.0	13	6.1	47	24.5	27	26.5	143	100
**Mixed†**	6	2.1	5	1.5	5	1.6	9	1.8	8	3.5	26	6.0	11	5.0	31	10.9	6	2.6	4	1.9	6	3.1	1	1.0	118	100
**Total**	287	100	325	100	322	100	512	100	229	100	431	100	218	100	284	100	230	100	214	100	192	100	102	100	3346	100

* Genotype analysis was performed on 3,346 faecal samples since 528 samples were partially or not genotyped.

† Other and mixed genotypes are listed in the Box.

Box. Rotavirus G-P combinations of other genotypes and mixed genotypes, Greece, 2008–2020

Other genotypes

Reassorted common human strains: G1P[4], G2P[8], G3P[4], G4P[4], G9P[4]

Reassortment between human and animal strains or strains with possible animal origin: G1P[9], G2P[10], G2P[6], G3P[9], G4P[10], G4P[14], G4P[6], G4P[9], G6P[14], G6P[9], G8P[14], G8P[8], G9P[10], G9P[9], G10P[4], G10P[8], G12P[11], G12P[4], G12P[6], G12P[9]

Mixed genotypes

More than one G-type: G1+G2P[4], G1+G3P[9], G1+G4P[8], G1+G9P[8], G1+G12P[8], G2+G4P[4], G2+G4P[8], G4+G12P[4], G4+G12P[8], G4+G9P[8], G8+G9P[8]

More than one P-type: G1P[8]+P[4], G10P[8]+P[10], G2P[8]+P[4], G2P[8]+P[10], G3P[8]+P[4], G4P[8]+P[4], G4P[8]+P[9], G9P[8]+P[6]

More than one G- and P-type: G2+G4P[8]+P[4], G4+G1P[8]+P[6]
